# The Latitudinal Patterns of Leaf and Soil C:N:P Stoichiometry in the Loess Plateau of China

**DOI:** 10.3389/fpls.2019.00085

**Published:** 2019-03-18

**Authors:** Zhao Fang, Dong-Dong Li, Feng Jiao, Jing Yao, Hao-Tian Du

**Affiliations:** ^1^State Key Laboratory of Soil Erosion and Dryland Farming on the Loess Plateau, Institute of Soil and Water Conservation, Northwest A&F University, Yangling, China; ^2^Institute of Soil and Water Conservation, Chinese Academy of Sciences and Ministry of Water Resources, Yangling, China; ^3^University of Chinese Academy of Sciences, Beijing, China

**Keywords:** ecological stoichiometry, climatic factors, herb community, vegetation types, leaves, soil, Loess Plateau

## Abstract

Understanding the spatial patterns and the driving factors of plant leaf and soil stoichiometry are critical for improving the parameterization of future ecological models and to predict the responses of ecosystems to environmental changes. This study aimed to determine how the latitudinal patterns of leaf and soil C:N:P stoichiometry are affected by climate and vegetation types in the dryland ecosystems. The concentrations of leaf C, N, and P in herb community as well as soil nutrient concentrations along a 500–km–long latitudinal gradient in Northern Shaanxi of the Loess Plateau, were measured. The results showed that the soil C, N, P and C:N:P ratios at all three depths (0–10, 10–20, and 20–40 cm) showed significant latitudinal trends (except for soil C:N ratios) (*P* < 0.01). In general, the soil C, N and C:N:P ratios decreased exponentially while soil P increased first and then decreased with the latitude. The soil C, N, C:P, and N:P ratios at all three depths (0–10, 10–20 and 20–40 cm) were positively correlated with MAT and MAP (*P* < 0.05), while soil P and C:N ratios at all three depths were weakly correlated with MAT and MAP (*P* > 0.05). In addition, leaf C:N:P stoichiometry was significantly correlated with the latitude, MAT, and MAP (except for N:P ratios) (*P* < 0.01), such that, leaf C, C:N, and C:P ratios decreased as the latitude increased and MAT and MAP decreased, and leaf N, P concentrations increased as the latitude increased and MAT and MAP decreased, while leaf N:P ratios were weakly correlated with the latitude, MAT, and MAP (*P* > 0.05). Furthermore, the leaf C:N:P stoichiometry of herbaceous communities was related to the soil properties (except for soil P), and we found that the C:P ratios between the soil and leaves were strongly correlated. Compared with the global scale, the relatively high N:P ratios indicated that the vegetation growth of the herb community in the dryland of the Loess Plateau was more susceptible to P limitation.

## Introduction

The terrestrial ecosystem consists of above- and belowground ecological components, and their strong interactions greatly affect the material cycle and energy flow in the terrestrial ecosystem (Wardle and Peltzer, 2007). In general, plants on the ground affect the soil properties by inputting litter and root exudates, while the intense activity of the microorganisms in the soil provides the mineralized nutrients and suitable environmental conditions needed for plant growth (Lambers et al., 2009; van der Heijden et al., 2010). A considerable body of evidence has suggested that the aboveground and belowground ecological components of ecosystems are tightly linked and are susceptible to environmental factors. Besides, the feedback between the above- and belowground components have important implications for the community structure and the ecosystem function (Bardgett et al., 2005; van der Putten et al., 2009; Laurel, 2013).

Ecological stoichiometry is a discipline that studies the balance of the various elements that are required by organisms and provides new perspectives for understanding the ecosystem structure and processes (Elser et al., 2000b; Yang and Luo, 2011). As essential components of organisms, carbon, nitrogen, and phosphorus play a vital role in studying nutrient cycling and the ecosystem structure and function (Koerselman, 1996; Sterner and Elser, 2002). Furthermore, the nutrient cycling of carbon, nitrogen, and phosphorus in the plant-soil system would tightly couple (Elser et al., 2000b). Nitrogen and phosphorus are generally considered to be the main limiting elements for terrestrial ecosystem productivity (Güsewell, 2010). Leaf N:P ratios have been a practical and important tool for detecting vegetation composition, dynamics and nutrient limitation in different ecosystems (Koerselman, 1996; Cernusak et al., 2010). Recently, the geographical pattern of plant and soil nutrient elements and its relationship with environmental factors has been studied extensively at global and regional scales (Reich et al., 1999; Han et al., 2005; He et al., 2006; Thompson et al., 2010; Wright et al., 2010; Wang et al., 2015), but the results have been inconsistent. For example, Tian et al. (2010) and Han et al. (2005) found that the C:N ratios of leaf, litter, and mineral soil in forests are well constrained at a national scale in China. Reich and Oleksyn (2004) revealed that global leaf N and P concentrations increased and N:P ratios decreased with increasing latitude (or decreasing mean annual temperature, MAT). In contrast, Han et al. (2005) and Ren et al. (2007) revealed similar research findings in China at the regional scale, but the differences were that N:P ratio was weakly correlated with the latitude and the MAT. In addition, Zheng and Shangguan (2007) found that C:N and C:P ratios in the leaves of the Loess Plateau were not related to the latitude and the MAT, but N:P ratio increased with the increasing latitude and was not correlated with MAT. Furthermore, Chen et al. (2016) found that the soil N and P concentrations both decreased significantly with the increasing MAT and the mean annual precipitation (MAP), while the soil N:P ratio did not vary in a systematic way with the latitude. Han et al. (2005) and Zheng and Shangguan (2007) concluded that this inconsistency may be attributed to the different research scales. So far, very little work has been done at the regional scale, especially in the Loess Plateau. To further confirm biogeographic patterns of leaf and soil nutrients at regional scales are more and more important for increasing the basic data of relevant research in the Loess Plateau and providing new materials to solve that inconsistent views.

The Loess Plateau in China, with an area of 6.2 km × 10^5^ km, is famous for having the highest rate of erosion in the world (Fu et al., 2000). For this reason, the Chinese government has launched a series of nationwide conservation projects to improve this dilemma, which is caused by the population pressure that has been increasing since the last century (An et al., 2013). These projects, such as the “Grain for Green” project, will inevitably have a major impact on regional soil nutrient cycling, and will in turn cause changes in the surface vegetation. Notably, the natural environment of the Loess Plateau varies greatly, and features such as rainfall, temperature, soil, and vegetation show regular changes from southeast to northwest (Yamanaka et al., 2014). This climatic variation at a regional scale along latitudinal gradients provides an excellent natural laboratory for investigating the formation mechanism and the relationship of the spatial distribution pattern of plant leaves and soil elements. According to the hydro-thermal conditions of the Loess Plateau, Cheng and Wan (2002) classified the vegetation of the Loess Plateau into five vegetation subzones: the forest zone (FO), the forest steppe zone (FS), the steppe zone (ST), the desert steppe, and the desert zone. Differences in the environmental conditions (temperature, moisture, etc.) are often linked to varying latitudes and, in turn could be used to explain the distribution of vegetation types across the Loess Plateau. In recent years, considerable research efforts have been devoted to surveying the effects of different vegetation restoration years, succession stages, and land use on the soil and plant ecological stoichiometry characteristics of the Loess Plateau (Jiao et al., 2011; Chai et al., 2015; Zhao et al., 2015). However, there are still few studies on the relationships between the soil and plant C:N:P stoichiometry and the latitudinal patterns of leaf and soil C:N:P stoichiometry.

In this study, a field survey was conducted on 15 subplots of different vegetation zones in the Loess Plateau to examine the changes in the soil and in the plant C:N:P stoichiometry and their relationships along latitudinal gradients, to reveal the limiting conditions of nutrient restrictions and provide a reference and basis for ecological restoration on the Loess Plateau. Our objectives were: (1) to determine the latitudinal patterns of leaf and soil C:N:P stoichiometry; (2) to quantify the relationships of the spatial variations of leaf and soil nutrients with climatic variables (MAT and MAP); (3) to quantify the relationships of the leaf nutrients traits with the soil nutrients traits; (4) to compare the patterns of leaf nutrients in the Loess Plateau with those of other scales.

## Materials and Methods

### Study Sites

**FIGURE 1:**
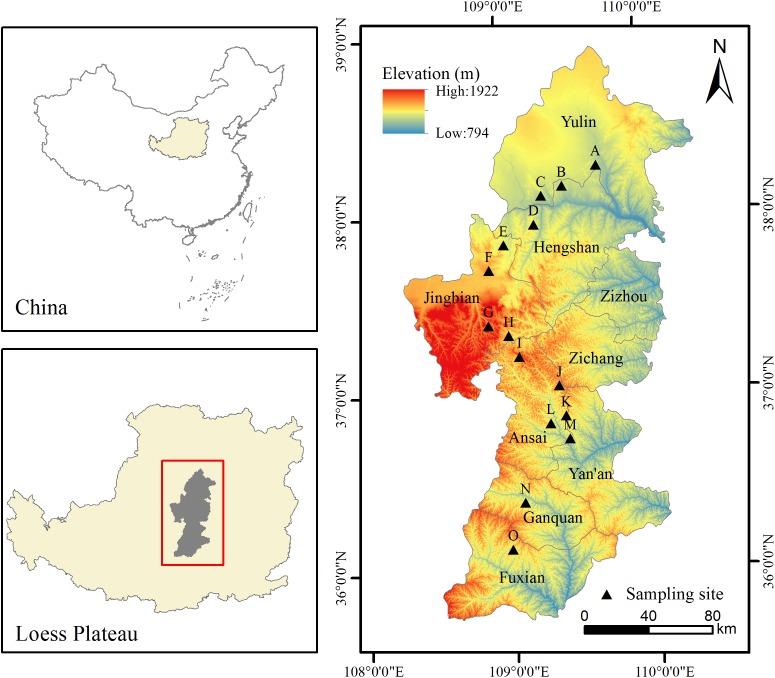
Sample arrangement diagram of study area.

**FIGURE 2:**
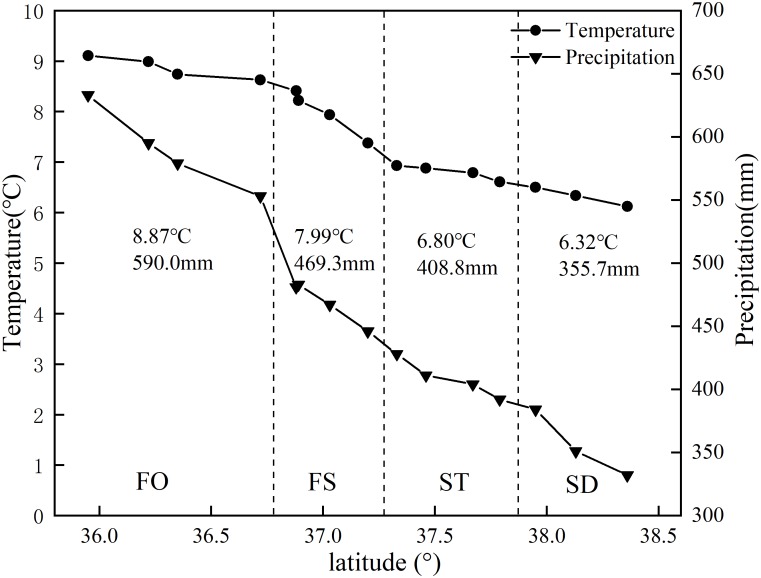
Climatic features of the sampling sites.

This study was conducted in fifteen sites along a 500–km–long latitudinal gradient in Northern Shaanxi of the Loess Plateau, which were located in the FO, FS, ST, and steppe-desert zone (SD), consistent with previous studies (Yamanaka et al., 2014) (Figure 1). The study area is located at N35°95′–38°36′ and E107°97′–109°87′ and belongs to the temperate zone. All sites were selected on flat terrain away from the human activity area, with minimal microclimate differences caused by the microtopography and grazing disturbance. This area was characterized by a temperate semi-arid climate with an annual mean temperature ranking of 8.87°C >7.99°C >6.80°C > 6.32°C and a MAP ranking of 590.0 mm > 469.3 mm > 408.8 mm > 355.7 mm for the four vegetation type zones (FO, FS, ST, and SD) (Figure 2) (1990–2010 data), which occurred during May to October in the growing season and most intensely in fall. The elevation of the study area ranged from 1,015 to 1,600 m above sea level. The zonal soil in this region was classified as Loessoal soils according to the Genetic Soil Classification of China (Shi et al., 2010). In this area, the main types of herb species were dominated by *Bothriochloa ischaemum*, *Stipa bungeana*, *Cleistogenes caespitosa*, *Lespedeza davurica*, *Astragalus melilotoides*, *Artemisia sacrorum*, *Heteropappus altaicus*, *Potentilla tanacetifolia*, etc.

### Soil and Plant Sampling and Analyses

Each vegetation zone type was represented by three to four subplots (30 m × 30 m); in total, there were 15 subplots. The spatial geographical coordinates (longitude and latitude) and altitude of each site were obtained by GPS. The basic information of the sample plots is shown in Table 1. Five 1 m × 1 m quadrats were surveyed at random in each subplot to represent the plot heterogeneity. Apparently, the species diversity and productivity of herb communities decreasing with the environmental gradients are shown in Table 2. And 23 herbaceous species (nine families) were selected based on the following criteria: the target species should be the dominant species and relatively abundant at each site. We selected healthy and mature leaves for each target species and collected them for about 30 g to bring them back to the laboratory in every 1 m × 1 m quadrat at the community level as well as in the community characteristics survey. The plant samples were dried at 75°C for 72 h to the appropriate constant mass.

**Table 1:** Descriptions of the sampling site.

Vegetation zone	Site	Latitude	Slope/(°)	Altitude/(m)	Target dominant species	Position
FO	A	35.95	28	1100	*Stipabungeana; Rubia cordifolia; Phragmites australis; Taraxacum mongolicum*	Southern Yanan
	B	36.22	17	1105	*Stipabungeana; Dracocephalum moldavica; Patrinia scabiosaefolia; Phragmites australis*	
	C	36.35	13	1015	*Stipabungeana; Lespedeza davurica; Taraxacum mongolicum; Incarvillea sinensis*	
	D	36.72	21	1100	*Stipabungeana; Lespedeza davurica; Taraxacum mongolicum; Phragmites australis*	
FS	E	36.88	19	1300	*Stipabungeana; Melilotus officinalis; Artemisia gmelinii; Lespedeza davurica*	Northern Yanan and Southern Ansai
	F	36.89	11	1330	*Stipabungeana; Melilotus officinalis; Saussurea amurensis; Lespedeza davurica*	
	G	37.03	15	1300	*Lespedeza davurica; Artemisia gmelinii; Bidens pilosa*	
	H	37.2	8	1277	*Artemisia gmelinii; Phragmites australis; Stipabungeana; Heteropappus altaicus*	
ST	I	37.33	11	1500	*Stipabungeana; Artemisia capillary; Dracocephalum moldavica; Lespedeza davurica*	Central Ansai and Southern Jingbian
	J	37.46	16	1500	*Stipabungeana; Lespedeza davurica; Artemisia desertorum; Scorzoneradivaricata*	
	K	37.67	12	1600	*Polygala tenuifolia; Erodium stephanianum; Dracocephalum moldavica; Vicia sepium*	
	L	37.79	12	1400	*Artemisia ordosica; Stipabungeana; Setaria viridis; Artemisia scoparia*	
SD	M	37.95	23	1100	*Artemisia desertorum; Heteropappus altaicus; Artemisia scoparia; Lespedeza davurica*	Northern Jingbian and Southern Yulin
	N	38.13	27	1148	*Artemisia desertorum; Artemisia scoparia; Astragalus adsurgens*	
	O	38.36	29	1205	*Artemisia desertorum; Artemisia scoparia; Lespedeza davurica*	


*FO, Forest zone; FS, Forest steppe zone; ST, Steppe zone; SD, Steppe-desert zone.*

**Table 2:** The species diversity index of the herb communities in different vegetation zones.

Vegetation zone	Species number	Shannon-Wiener index	Pielou index	Community productivity (g/m^2^)
FO	11.5 ± 1.21a	2.71 ± 0.21a	0.87 ± 0.02a	178.80 ± 14.7ab
FS	8.75 ± 0.98bc	2.47 ± 0.14b	0.76 ± 0.03ab	191.21 ± 13.5a
ST	9.55 ± 1.03b	2.43 ± 0.16ab	0.74 ± 0.05ab	144.86 ± 10.4b
SD	6 ± 0.77d	2.23 ± 0.25c	0.54 ± 0.11c	98.35 ± 9.5c


*Values are the means ± SE in the above table. Within each column, means with different letters are significantly different based on ANOVA and LSD (*P* < 0.05).*

Five soil samples were collected randomly from each quadrat with use of a soil drilling sampling corer (9 cm in diameter) in accordance with serpents sampling and litter was cleared before soil sampling. The samples in the soil layer were collected at intervals of 0–10, 10–20, and 20–40 cm with a soil core (5 cm in diameter), and the samples from the same layer were mixed into a composite sample. All soil samples were naturally air-dried in the laboratory and were sieved thoroughly through a 2–mm screen, and other debris were removed by hand for an analysis of the soil chemical properties. The organic C concentrations of the soil and plant samples were analyzed by the classical potassium dichromate external heating method, and the total N concentrations of the soil and plant samples were measured via the semi-micro Kjeldahl method; in addition, the total P concentration was analyzed colorimetrically by the ammonium molybdate method (Bao, 2000).

### Statistical Analyses

All variables were described by the mean and standard error (SE), and SPSS 18.0 software (SPSS Inc., Chicago, IL, United States) was used for the statistical analysis. All of the data were checked for the normality and homogeneity of the variances before applying the parametric tests. One-way ANOVA and multiple comparisons (LSD) were used to test the differences between the plant leaves and the soil nutrients and the stoichiometric characteristics in different vegetation zones. Regressions were linear for the latitudinal patterns of leaf C:N:P stoichiometry, while nonlinear fits were used for the latitudinal patterns of soil C:N:P stoichiometry. The Pearson coefficient test was used to measure the correlation between leaf and soil C:N:P stoichiometry and climatic variables. The figures were conducted using Origin 9.0. The plant leaf and soil nutrients concentrations were expressed as mg⋅g^-1^ on a dry mass basis, and all of the C:N:P stoichiometric ratios in leaves and soil were mass ratios.

## Results

### Patterns of Soil C, N, and P Concentrations and Ratios Along Latitudinal Gradients

**FIGURE 3:**
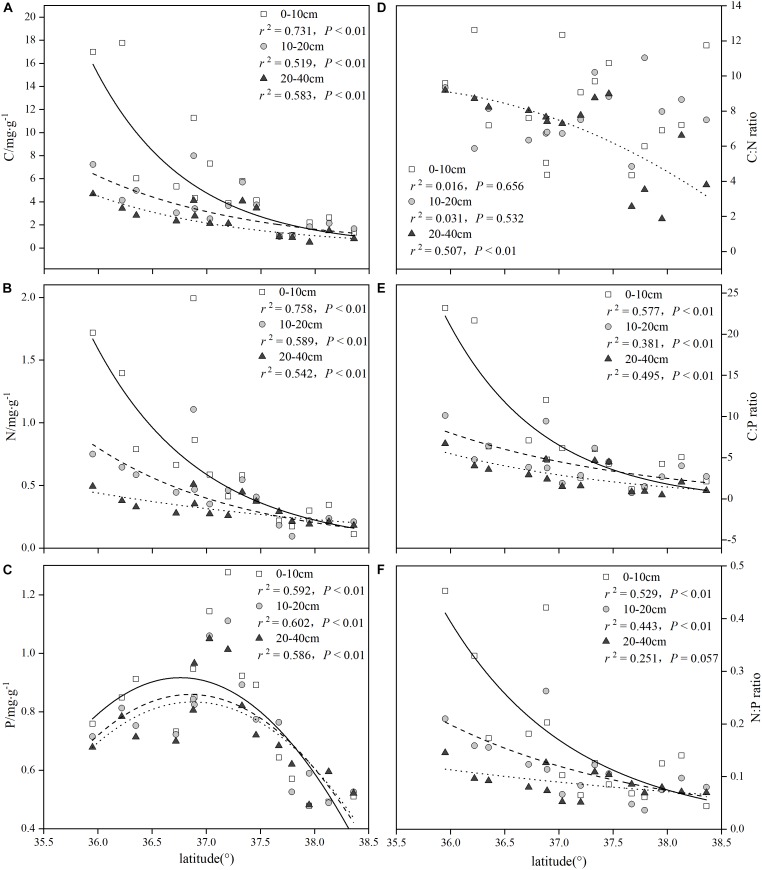
Summary of the regression analyses for soil C:N:P stoichiometry along latitudinal gradients.

As seen from parts A and B of Figure 3, the concentrations of the soil C and the soil N at all three depths tended to decline exponentially along the latitudinal gradients, and all of the regression equations reached significant levels (*P* < 0.01). In contrast, with increasing latitude, the concentrations of soil P in the three soil layers were significantly diminished by the binomial approach (*P* < 0.01) (Figure 3C). The patterns for the soil C:N ratios showed poor performance; however, only the 20–40 cm soil layers significantly decreased along the latitudinal gradients (*P* < 0.01) (Figure 3D). The patterns for the soil C:P and N:P ratios were similar to those of the soil C and N concentrations (Figure 3E,F), and an exponential regression equation was suitable for the trend of the soil C:P and N:P ratios variations in all three soil depths, while the soil N:P ratio (20–40 cm) was not noticeable in the exponential regression (*P* > 0.05).

To further explore this difference, we compared the differences in the soil nutrients between the four different vegetation zones in the 0–40 cm soil layer (Table 3). The soil C and N concentrations of the three soil layers in the FO were the highest, while the soil P concentrations of the three soil layers in the FS were the highest among the four vegetation zones. The concentrations of total soil C and total soil N in the 0–10 cm soil layer varied with a ranking of FO > FS > ST ≈ SD; similarly, a ranking of FO > FS ≈ ST > SD was presented in the 20–40 cm soil layer for the concentrations of the total soil C and the total soil N. Furthermore, the concentrations of total soil P in all three soil layers had the same varied trend in all four vegetation zones: FS > FO ≈ ST > SD. The C:N ratio of 20–40 cm and the N:P ratio of 0–10 cm showed obvious differences in the four vegetation zones, but the ratios of C:P showed a significant difference in the 0–10 and 20–40 cm soil layers of the four vegetation zones.

**Table 3:** The concentrations of soil nutrients and the soil ecological stoichiometry.

Soil layer	Vegetation zone types	C content (g/kg)	N content (g/kg)	P content (g/kg)	C:N ratio	C:P ratio	N:P ratio
0–10 cm	FO	11.53 ± 3.38a	1.14 ± 0.25a	0.81 ± 0.04b	9.57 ± 1.16a	14.31 ± 4.26a	1.42 ± 0.33a
	FS	6.70 ± 1.70ab	0.96 ± 0.36ab	1.05 ± 0.10a	8.13 ± 1.74a	6.61 ± 1.89ab	0.99 ± 0.40ab
	ST	3.04 ± 1.16b	0.34 ± 0.09b	0.76 ± 0.09b	8.12 ± 1.41a	3.65 ± 1.10b	0.42 ± 0.72b
	SD	2.06 ± 0.38b	0.25 ± 0.07b	0.49 ± 0.09c	8.98 ± 1.46a	4.20 ± 0.82b	0.52 ± 0.15ab
10–20 cm	FO	4.86 ± 0.89a	0.61 ± 0.06a	0.75 ± 0.22b	7.87 ± 0.75a	6.52 ± 1.30a	0.81 ± 0.89a
	FS	4.41 ± 1.22a	0.60 ± 0.17a	0.96 ± 0.07a	7.42 ± 0.18a	4.83 ± 1.59a	0.66 ± 0.22a
	ST	2.89 ± 1.14a	0.31 ± 0.10ab	0.74 ± 0.77b	9.08 ± 1.28a	3.64 ± 1.19a	0.39 ± 0.11a
	SD	1.89 ± 0.14a	0.22 ± 0.01c	0.53 ± 0.30c	8.44 ± 0.31a	3.56 ± 0.41a	0.42 ± 0.03a
20–40 cm	FO	3.33 ± 0.51a	0.37 ± 0.46a	0.72 ± 0.02b	8.91 ± 0.24a	4.65 ± 0.78a	0.52 ± 0.07a
	FS	2.78 ± 0.47ab	0.35 ± 0.57ab	0.96 ± 0.54a	7.96 ± 0.10ab	3.02 ± 0.72ab	0.38 ± 0.09a
	ST	2.36 ± 0.83ab	0.33 ± 0.05ab	0.71 ± 0.04b	6.50 ± 1.58ab	3.17 ± 1.00ab	0.46 ± 0.05a
	SD	0.94 ± 0.29c	0.19 ± 0.01c	0.53 ± 0.03c	4.75 ± 1.29c	1.72 ± 0.43b	0.37 ± 0.02a


*Means with different small letters were significantly different at the 0.05 level for different vegetation zones at the same soil layer.*

### Patterns of Leaf C, N, and P Concentrations and Ratios Along Latitudinal Gradients

**FIGURE 4:**
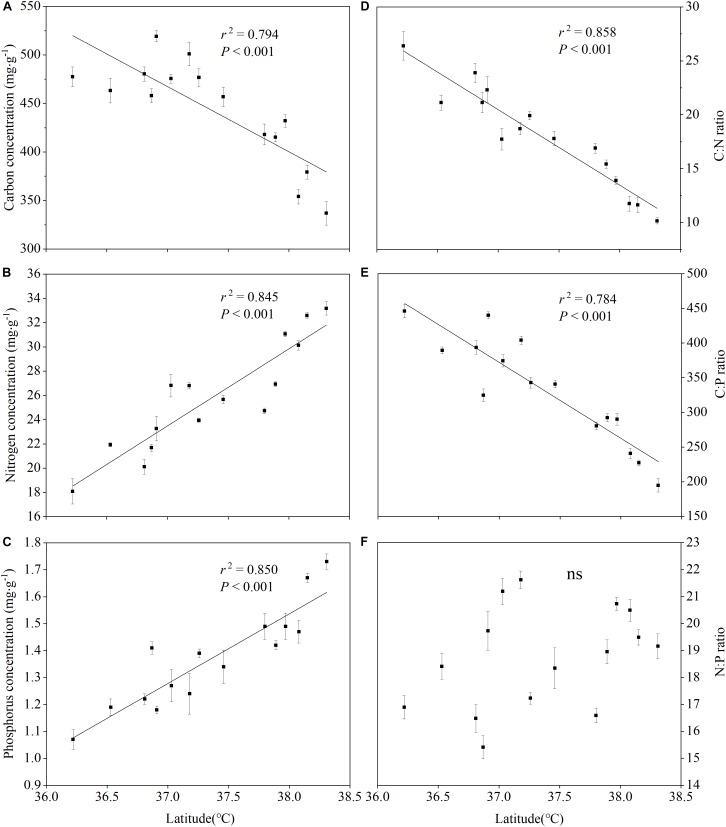
Summary of the regression analyses for leaf C:N:P stoichiometry along latitudinal gradients.

The leaf C, N, and P nutrient patterns in Figure 4 show that the latitudinal gradient had a significant effect on the leaf total C, total N and total P concentrations. There was an obviously different trend between the leaf C concentrations and the leaf N, P concentrations along the latitudinal gradient. With the increase of latitude, the leaf C concentrations remarkably decreased (*r*^2^ = 0.794, *P* < 0.001) (Figure 4A), while the leaf N, P concentrations apparently increased (*r*^2^ = 0.845, *P* < 0.001; *r*^2^ = 0.850, *P* < 0.001) (Figure 4B,C). Furthermore, the patterns for the leaf C:N ratios (Figure 4D) and C:P ratios (Figure 4E) were similar to that for the leaf C concentrations (Figure 4A). The relationship between the leaf N:P and the latitude, however, was not significant (*r*^2^ = 0.085, *P* = 0.723) (Figure 4F). Furthermore, our analysis of variance on the leaf nutrients and the C:N:P characteristics stoichiometry was consistent with the observation results above (Table 4).

**Table 4:** The concentrations of the leaf nutrients and the leaf ecological stoichiometry.

Vegetation zone types	C content (g/kg)	N content (g/kg)	P content (g/kg)	C:N ratio	C:P ratio	N:P ratio
FO	469.70 ± 5.41a	20.45 ± 0.88c	1.22 ± 0.07c	23.1 ± 1.27a	388.46 ± 24.85a	16.80 ± 0.62c
FS	493.06 ± 10.49a	25.20 ± 0.93b	1.27 ± 0.04bc	19.67 ± 0.99b	390.31 ± 20.72a	19.94 ± 0.99a
ST	430.47 ± 9.50b	27.10 ± 1.40b	1.44 ± 0.04b	16.00 ± 0.85c	300.92 ± 13.56b	18.65 ± 0.85ab
SD	356.76 ± 12.30c	31.96 ± 0.93a	1.62 ± 0.08a	11.2 ± 0.51d	220.91 ± 13.65c	19.71 ± 0.40a


*Means with different small letters were significantly different at the 0.05 level for different vegetation zones.*

### Relationships Between the Soil Nutrients and the Leaf Stoichiometry

**FIGURE 5:**
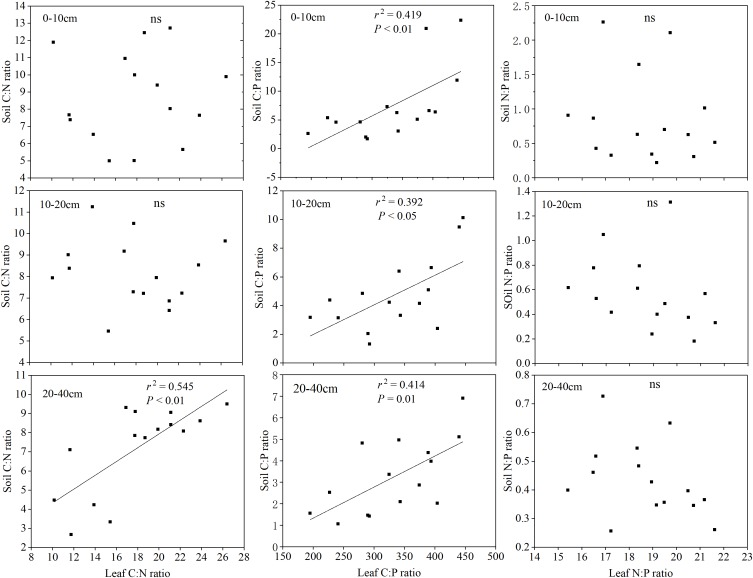
Relationships between the soil and leaf C:N:P stoichiometry.

There were significant differences in the correlations among different soil layer C, N, P concentrations, the leaf C, N, P concentrations and the C:N:P stoichiometric characteristics (Table 5). Overall, the different soil layers of the C, N, P concentrations were positively correlated with the leaf C concentrations, while being negatively correlated with the leaf N, P concentrations (except the soil P concentrations). The correlation between the leaf N:P and the different soil layer C, N, P nutrient concentrations was weakly (*P* > 0.05), while the different soil layer C, N, P nutrient concentrations and leaf C:N, and C:P stoichiometric characteristics showed a significant or extremely significant positive correlation. More interestingly, a significantly positive correlation with the soil total phosphorus concentration in different soil layers, the leaf C concentrations (*P* < 0.01), and the leaf N, P concentrations were not significant (*P* > 0.01). The correlation analysis between the soil and leaf stoichiometric characteristics in Figure 5 shows that the soil C:N ratio of 20–40 cm was significantly correlated with the leaf C:N ratio (*P* < 0.01), and the ratios of soil C:P at all three depths were significantly correlated with the leaf C:P ratio. In contrast, there was no significant correlation between the soil N:P ratios at all three depths and the leaf N:P ratio.

**Table 5:** Correlation coefficients between the leaf stoichiometry characteristics and the soil nutrients.

	0–10 cm			10–20 cm			20–40 cm		
	Soil C	Soil N	Soil P	Soil C	Soil N	Soil P	Soil C	Soil N	Soil P
Leaf C	0.567*	0.671*	0.782**	0.653*	0.710**	0.723**	0.685*	0.675*	0.775^∗∗^
Leaf N	–0.668*	–0.648*	–0.511	–0.691*	–0.696*	–0.48	–0.757**	–0.630*	–0.36
Leaf P	–0.736**	–0.637*	–0.458	–0.684*	–0.714**	–0.514	–0.712**	–0.772**	–0.456
Leaf C:N	0.749**	0.771**	0.609	0.777**	0.784**	0.532*	0.791**	0.746**	0.468
Leaf C:P	0.756**	0.823**	0.659*	0.755**	0.794**	0.619*	0.758**	0.771**	0.629*
Leaf N:P	–0.127	–0.078	0.053	–0.267	–0.194	–0.007	–0.338	–0.209	0.223


*^∗^Correlation is significant at the 0.05 level (2-tailed), ^∗∗^Correlation is significant at the 0.01 level (2-tailed).*

### Relationships Between Leaf and Soil Nutrient Traits With Climatic Variables

Leaf C, C:N, and C:P ratios were positively correlated with MAT (*r* = 0.783, *P* < 0.01; *r* = 0.918, *P* < 0.01; *r* = 0.887, *P* < 0.01) and MAP (*r* = 0.693, *P* < 0.01; *r* = 0.922, *P* < 0.01; *r* = 0.820, *P* < 0.01), leaf N and P were negatively correlated with MAT (*r* = -0.876, *P* < 0.01; *r* = -0.871, *P* < 0.01) and MAP (*r* = -0.915, *P* < 0.01; *r* = -0.851, *P* < 0.01), while leaf N:P ratios were weakly correlated with MAT and MAP (*P* > 0.05). For soil nutrient traits, the soil C, N, C:P, and N:P ratios at all three depths (0–10, 10–20, and 20–40 cm) were positively correlated with MAT (*r* = 0.592–0.842, *P* < 0.05) and MAP (*r* = 0.592–0.814, *P* < 0.05), while the soil P and C:N ratios at all three depths were weakly correlated with MAT and MAP (*P* > 0.05) (Table 6).

**Table 6:** Correlation coefficients between leaf, soil nutrient traits, and climatic variables in the Loess Plateau of China.

Leaf	MAT	MAP	0–10cm Soil	MAT	MAP	10–20cm Soil	MAT	MAP	20–40cm Soil	MAT	MAP
C	0.783**	0.693**	C	0.842**	0.814**	C	0.646**	0.619**	C	0.678**	0.672**
N	–0.876**	–0.915**	N	0.802**	0.734**	N	0.730**	0.649**	N	0.625*	0.604*
P	–0.871**	–0.851**	P	0.465	0.39	P	0.412	0.339	P	0.427	0.308
C/N	0.918**	0.922**	C/N	0.066	0.131	C/N	–0.298	–0.182	C/N	0.335	0.526
C/P	0.877**	0.820**	C/P	0.778**	0.774**	C/P	0.592*	0.592*	C/P	0.629*	0.666**
N/P	–0.342	–0.464	N/P	0.759**	0.724**	N/P	0.697**	0.640*	N/P	0.434*	0.492*


*^∗^Correlation is significant at the 0.05 level (2-tailed), ^∗∗^Correlation is significant at the 0.01 level (2-tailed).*

## Discussion

### The Latitudinal Patterns of Soil C:N:P Stoichiometry in Response to Climate

In the current study, the soil C and soil N concentrations in different soil layers showed an exponential decrease as latitude increased, which was consistent with previous findings (Wu et al., 2003; Yang et al., 2007; Zhu et al., 2013). Nitrogen in the soil mainly depends on the accumulation and decomposition of organic matter, which was positively correlated with the soil organic matter, and consequently, the soil N and soil C showed consistent spatial distribution patterns. A possible explanation for the pattern of the C:N ratios were weakly correlations with the latitude in that the trends for the soil N with respect to the latitude were general similar to the trends for soil C, which also verified the relative stability of the soil C:N ratios in different ecosystems (Tian et al., 2010). Previous findings have suggested that the concentrations of soil C and soil N were determined by the relative size of the input and output quantities of soil organic matter and nitrogen (Trumbore et al., 1996); meanwhile, the climate (temperature and precipitation) and the soil formation are main factors that affect the characteristics of the soil ecological chemometrics (Tian et al., 2010). The results of correlation analysis showed that the concentrations of soil C and N in all three soil depths (0–10, 10–20, and 20–40 cm) were significantly correlated with MAP and MAT (*P* < 0.05) (Table 6), which indicated that the soil C and N were influenced by biological action under natural conditions. In this area, the temperature and rainfall showed a significant downward trend with increasing latitude, which resulted in high P leaching rate and weathering rate. At the same time, the high species diversity and productivity of the FO and the FS maintained relatively high soil C and N concentrations (Table 2), which gave these regions higher C:P and N:P ratios. However, the dry and cool climate regime in the ST and in the SD resulted in low species diversity and productivity (Table 2), lower soil C and N concentrations and low P leaching rate. Hence, the soil organic carbon and the total nitrogen displayed a decreasing trend with the latitudinal gradients. In addition, this was demonstrated in a number of studies that showed that the soil C and N concentrations decreased with the increase in soil depth (Drahorad et al., 2013; Gao and Cheng, 2013).

In contrast, the spatial variability of soil P is relatively smaller than that of soil C and N in our study. As mentioned above, high P leaching rate in the FO and the FS resulted in decreasing of soil P concentrations and high productivity and abundant litter transported more nutrients for the soil, promoting accumulation of the soil C, N, and P (Muller, 2014). Lower productivity in the ST and in the SD led to reduced nutrient input, which gave these regions relatively low soil P concentrations. Based on an inventory data set of 2,384 soil profiles, Tian et al. (2010) found that the soil P concentrations were affected by a series of factors such as the parent material, climate, biology, and geochemical processes in the soil. Our previous studies suggested that the soil P concentrations at all three depths were weakly correlated with MAT and MAP (*P* > 0.05) which this weak relationship probably be attributed to the smaller scale in this study.

### The Latitudinal Patterns of Leaf C:N:P Stoichiometry in Response to Climate

Considerable research efforts have been devoted to investigate the geographical pattern of plant nutrient elements and its relationship with environmental factors at local, regional, or global scales. The leaf C concentrations sharply decreased with the increasing latitude. A better explanation for this decline is that the plants overcame stress conditions (the temperature increase aggravates soil aridity with the decline of latitude) by increasing their structural carbon compounds (cellulose, lignin, cutin, and waxes) (Bussotti et al., 2000). Reich and Oleksyn (2004) revealed that global leaf N and P concentrations increased and N:P ratios decreased with increasing latitude (or decreasing mean annual temperature, MAT). The concentrations of leaf N and P showed an increasing trend along the latitudinal gradients, which is a phenomenon that can be explained well by the Temperature-Plant Physiological Hypothesis (Reich and Oleksyn, 2004). In contrast, Han et al. (2005) and Ren et al. (2007) revealed similar research findings in China at the regional scale, but the differences were that N:P ratio was weakly correlated with the latitude and the MAT. In addition, Zheng and Shangguan (2007) found that C:N and C:P ratios in the leaves of the Loess Plateau were not related to the latitude and the MAT, but N/P increased with the increasing latitude and was not correlated with MAT. In this study, leaf C, C/N, and C/P ratios decreased as the latitude increased and MAT and MAP decreased, and leaf N, P concentrations increased as the latitude increased and MAT and MAP decreased, while leaf N/P ratio was unrelated to the latitude, MAT. and MAP (Figure 4 and Table 6). Possible reasons for this inconsistency may be attributed to the difference of research scales and sample size. Our study had a smaller geographical range with its latitudes ranging from 36 to 39°N and its sample size was much smaller than that in other scholars’ studies.

Furthermore, to deeply reveal the level of the leaf nutrients and the leaf C:N:P characteristics of the herbaceous vegetation of the Loess Plateau, we compared the results of this study with other scholars’ findings and found that the leaf C concentrations of herbaceous plants in the Loess Plateau were significantly lower than that of the average global level of Elser et al. (2000a), which was consistent with the results of Zheng and Shangguan (2007) for the leaf C concentrations in the Loess Plateau. Furthermore, the leaf N concentrations of herbaceous plants on the Loess Plateau were significantly higher than the level at the global scale (Reich et al., 1999; Elser et al., 2000a), but were also slightly higher than that found on the Loess Plateau by Zheng and Shangguan (2007). Herbs generally absorb highly mobile mineral nitrogen (such as nitrate nitrogen and ammonium nitrogen), which can be used to improve the level of leaf N nitrogen concentrations directly. The influence of hydro-thermal factors increased the mineralization rate of soil N on the Loess Plateau, thereby increasing the leaf N concentrations of herbaceous plants (Heerwaarden et al., 2010). In recent years, the soil available nitrogen increased due to the increase of nitrogen deposition (Galloway et al., 2008), and it also promoted the leaf N concentrations of herbaceous plants. Furthermore, the leaf P concentrations of herbaceous plants on the Loess Plateau were significantly lower than that at the global scale (Reich et al., 1999; Elser et al., 2000a), but were also slightly lower than that at the Loess Plateau (Zheng and Shangguan, 2007) scale and at the China national scale (Han et al., 2005). This reveals that the P concentrations of herbaceous plants on the Loess Plateau scale and even on the China national scale were still kept at a fairly low level. Studies have showed that, serious soil erosion causes great losses of plant nutrients, which may lead to herbaceous plants on the Loess Plateau to be at low phosphorus levels (Zheng and Shangguan, 2007). In brief, compared with the global scale, the Loess Plateau is characterized by lower C, P concentrations and higher N concentrations for herbaceous leaves, which results in lower leaf C:N ratio and higher C:P and N:P ratios than those of the global scale.

### Relationships Between Leaf and Soil C:N:P Stoichiometry

Plant and soil, as different components in the biogeochemical cycle, are closely linked and interact with each other; however, a few examples have been reported to show how the concentrations of C, N, and P in soil were related to their concentrations in plants (Jobbágy and Jackson, 2001; Yu et al., 2010). Our results show that there was strong links between leaf C:N:P stoichiometry and soil properties (except for soil P), which is consistent with previous studies (Han et al., 2005; Zeng et al., 2016). Generally, the absorption and utilization of soil nutrients is the main purpose for the output of soil available nutrients. The soil and herbaceous plants achieve and maintain a balanced proportion of elements through the dynamic exchange of nutrient supply and demand. In this report, the soil P concentrations were not positively related to the leaf N, P concentrations, which confirmed to the results of Yang et al. (2018). According to the correlation analysis between the soil and leaf stoichiometric characteristics, we found that the soil C:P ratios in all three depths were significantly related to the leaf C:P ratios. This observation is mainly related to an extremely significant positive correlation between the soil P concentrations in all three depths and the leaf C concentrations (Table 5). Moreover, the dynamics of the plant leaf nutrients are primarily restricted by the soil P supply on the Loess Plateau, which we will discuss below. Furthermore, the soil C:N ratio of 20–40 cm was significant correlated with the leaf C:N ratios, probably because the soil C and N concentrations in the 0–10 and 10–20 cm soil layers varied greatly.

The leaf N:P ratios were used as an indicator of the N-limitation or P-limitation in the ecosystems; i.e., N:P ratios <14 suggest N limitation, and N:P ratios >16 suggest P limitation (Güsewell, 2010). In this study, the average N:P ratios of the four vegetation zones were all higher than 16 (Table 4), which also suggested an increase in the P limitation in the Loess Plateau compared with the results of a global scale study (Elser et al., 2000a; Reich and Oleksyn, 2004). Therefore, further research should pay more attention to the transformation of the soil nutrient-limited elements and the interactions among the C:N:P stoichiometry in the plant-soil systems, especially for large-scale vegetation restoration projects on the Loess Plateau.

## Conclusion

Our study provided a complete picture of the spatial patterns of the C, N, and P elements of leaves and the soil stoichiometry along the latitude of the Loess Plateau. The results suggested that the soil C, N, P, and C:N:P ratios at all three depths (0–10, 10–20, and 20–40 cm) showed significant latitudinal trends (except for soil C:N ratios) (*P* < 0.01). In general, the soil C, N, and C:N:P ratios decreased exponentially while soil P increased first and then decreased with the latitude. The soil C, N, C:P, and N:P ratios at all three depths (0–10, 10–20, and 20–40 cm) were positively correlated with MAT and MAP (*P* < 0.05), while soil P and C:N ratios at all three depths were weakly correlated with MAT and MAP (*P* > 0.05). In addition, leaf C:N:P stoichiometry was significantly correlated with the latitude, MAT, and MAP (except for N:P ratios) (*P* < 0.01), such that, leaf C, C:N, and C:P ratios decreased as the latitude increased and MAT and MAP decreased, and leaf N, P concentrations increased as the latitude increased and MAT and MAP decreased, while leaf N:P ratios were weakly correlated with the latitude, MAT, and MAP (*P* > 0.05). In addition, the leaf C:N:P stoichiometry of the herbaceous communities is related to the soil properties (except for soil P), and we found that the soil and plant C:P ratios are strongly related, which indicated strong links among the C:N:P stoichiometry in leaves and the soil properties. Compared with the vegetation of global scale, the vegetation of the Loess Plateau is more susceptible to P limitation.

## Data Availability

All datasets generated for this study are included in the manuscript and/or the supplementary files.

## Author Contributions

FJ contributed to study design, statistical analyses, and data management. D-DL, JY, and H-TD performed the experiments. ZF wrote the manuscript.

## Conflict of Interest Statement

The authors declare that the research was conducted in the absence of any commercial or financial relationships that could be construed as a potential conflict of interest.
